# Cancer burden among adolescents and young adults in relation to childhood adversity: a nationwide life-course cohort study of 1.2 million individuals

**DOI:** 10.1016/j.lanepe.2023.100588

**Published:** 2023-02-10

**Authors:** Naja Hulvej Rod, Jessica Bengtsson, Leonie K. Elsenburg, Megan Davies, David Taylor-Robinson, Samir Bhatt, Andreas Rieckmann

**Affiliations:** a Section of Epidemiology, Department of Public Health, University of Copenhagen, Denmark; b Institute of Public Health, University of Liverpool, UK; c School of Public Health, Imperial College London, UK

**Keywords:** Childhood adversity, Cancer incidence, Cancer mortality, Cancer case fatality, Health inequality

## Abstract

**Background:**

Childhood adversity such as poverty, loss of a parent, and dysfunctional family dynamics may be associated with exposure to environmental and behavioral hazards, interfere with normal biological functions, and affect cancer care and outcomes. To explore this hypothesis, we assessed the cancer burden among young men and women exposed to adversity during childhood.

**Methods:**

We undertook a population-based study using Danish nationwide register data on childhood adversity and cancer outcomes. Children who were alive and resident in Denmark until their 16th birthday were followed into young adulthood (16–38 years). Group-based multi-trajectory modelling was used to categorize individuals into five distinct groups: low adversity, early material deprivation, persistent material deprivation, loss/threat of loss, and high adversity. We assessed the association with overall cancer incidence, mortality, and five-year case fatality; and cancer specific outcomes for the four most common cancers in this age group in sex-stratified survival analyses.

**Findings:**

1,281,334 individuals born between Jan 1, 1980, and Dec 31, 2001, were followed up until Dec 31, 2018, capturing 8229 incident cancer cases and 662 cancer deaths. Compared to low adversity, women who experienced persistent material deprivation carried a slightly lower risk of overall cancer (hazard ratio (HR) 0.90; 95% CI 0.82; 0.99), particularly due to malignant melanoma and brain and central nervous system cancers, while women who experienced high adversity carried a higher risk of breast cancer (HR 1.71; 95% CI 1.09; 2.70) and cervical cancer incidence (HR 1.82; 95% CI 1.18; 2.83). While there was no clear association between childhood adversity and cancer incidence in men, those men who had experienced persistent material deprivation (HR 1.72; 95% CI 1.29; 2.31) or high adversity (HR 2.27; 95% CI 1.38; 3.72) carried a disproportionate burden of cancer mortality during adolescence or young adulthood compared to men in the low adversity group.

**Interpretation:**

Childhood adversity is associated with a lower risk of some subtypes of cancer and a higher risk of others, particular in women. Persistent deprivation and adversity are also associated with a higher risk of adverse cancer outcomes for men. These findings may relate to a combination of biological susceptibility, health behaviors and treatment-related factors.

**Funding:**

None.

**Research in context**

**Evidence before this study**

Cancer is the most common cause of disease-related deaths in adolescents and young adults, but the underlying causes remain understudied. Social adversity may affect cancer incidence and prognosis through various mechanisms including differential exposure to risk factors, biological vulnerability, differences in access to care, co-morbidities, and adherence and response to treatment. We did a comprehensive literature review in PubMed from inception to August 5, 2022, using the search terms (“adverse childhood events” or “bereavement” or “adversity” or “stressors” or [life change events]) and ([neoplasms] or “cancer”) in English or Danish. We identified articles assessing childhood adversity and its associations with cancer incidence, cancer mortality and cancer case fatality. Cancers occurring in adolescents and young adults differ from those occurring during childhood or in older age groups in terms of cancer types, tumor biology and prognosis. However, very few studies have assessed the impact of early life adversity on cancer burden among adolescents and young adults under 40 years using objective life course data.

**Added value of this study**

We use unselected annually updated nationwide register data covering 1.2 million Danish adolescents and young adults (ages 16–38) to assesses the complex relationship between childhood social and family-related adversities and cancer incidence (including the four most common cancer types by sex in this age group), cancer mortality, and 5-year case-fatality. We measure a comprehensive range of childhood adversity based on 12 different annual exposures between age 0 and 16 years over three dimensions: material deprivation, loss or threat of loss, and family dynamics. We show that childhood adversity is associated with a higher risk of breast and cervical cancer incidence among young women exposed to childhood adversity. For men there was no clear association between childhood adversity and cancer incidence, but young men who had experienced social adversity during childhood carried a disproportionate burden of cancer mortality and case fatality during adolescence or young adulthood compared to their peers.

**Implications of all the available evidence**

Adolescence and young adulthood are formative periods of life and a cancer diagnosis during these years can have a major impact on life trajectories. Exposure to persistent adversity during childhood is likely to create ongoing social vulnerability which amplifies the adverse consequences of a diagnosis of cancer at a young age. This calls for broader structural public health and policy interventions aimed at reducing the underlying social drivers creating childhood adversity in conjunction with clinical services that are responsive to the social needs of young cancer patients, providing support to ensure that vulnerable groups can achieve optimal outcomes from treatment.

## Introduction

Cancers occurring in adolescents and young adults contribute substantially to the global burden of disease,^1^ and cancer incidence appears to be trending upwards in young age groups.^2^^,^^3^ Adolescents and young adults’ cancers seem to differ from those occurring during childhood or in older age groups in terms of cancer types, tumor biology and prognosis,^3^^,^^4^ but the causes and consequences of cancers occurring in adolescents and young adults remain understudied. Adolescence and early adult life constitute a period with major life transitions that may negatively affect both the timeliness of the diagnosis and adherence to treatment,^5^ making it even more important to describe the cancer burden and its associated risk factors in this age-group separately.

Patients with cancer commonly voice the belief that life stress may have been a contributory cause for their disease,^6^ and, as a result, there is a longstanding scientific interest in the impact of early life adversity on cancer risk. Social adversity may affect cancer incidence and prognosis through various mechanisms including differential exposure to risk factors as well as differential vulnerability.^7^ Children who grow up in socially deprived families are generally more likely than their peers to be exposed to environmental hazards and behavioral risk factors including air pollution, smoking, and poor diet. At the same time, they may be more biologically susceptible to such hazards, as stressful experiences in early life can leave a long-lasting impact on emerging brain structure and thereby interfere with normal biological functions.^8^ Evidence from experimental studies suggests that the stress resulting from adverse circumstances can result in persistent activation of the biological stress response system, with associated immunosuppressive effects including lower levels of cytotoxic T-cells and natural-killer-cell activity, which are essential for immune surveillance of tumors.^9^ These mechanisms may be amplified by differences in support, access to care, co-morbidities, and adherence and response to treatment.

In line with these hypotheses, two recent reviews and meta-analyses have suggested a higher risk of cancer incidence associated with childhood adversity, including abuse and financial difficulties within the family.^10^^,^^11^ However, the results from the underlying individual studies were heterogeneous and all findings were based on retrospective reporting of adverse childhood events in adulthood, which is prone to reporting and survival bias. Only a few studies included individuals below the age of 40 years, and none of them specifically addressed cancers among adolescents and young adults. Furthermore, most studies were cross-sectional in design and the evidence from the few prospective studies was mixed.^12^, ^13^, ^14^, ^15^ More recently, we have shown a higher risk of cancer mortality among young adults exposed to high vs low adversity in a large prospective study of children followed from birth until 34 years of age.^16^ However, in a subset of these children followed-up until the age of 24 with detailed information on hospitalization patterns, cancer incidence was not associated with childhood adversity.^17^ Considering these mixed findings, combined with the heterogeneity of previous findings, there is a clear need for further investigations into the effect of childhood adversity on adolescent and young adult cancer incidence and cancer mortality.

Cancer is a heterogeneous group of diseases with different underlying etiology, and we hypothesize that childhood adversity measured through dimensions of material deprivation, loss or threat of loss and family dynamics will affect specific subtypes of cancer differently through an impact on behavioral factors (smoking, alcohol intake, physical activity, sun exposure), infections and environmental factors.^10^^,^^17^ We also hypothesize that childhood adversity may impact cancer survival through socially patterned differences in biological susceptibility, access to support and care, and treatment adherence. We use unselected nationwide data on 1.2 million individuals followed-up from age 16–38 to test these hypotheses. We address cancer incidence and cancer outcomes separately, capturing the latter using cancer specific mortality and five-year case fatality. We also assess the relation between childhood adversity and the four most common types of cancer in this age group among women (malignant melanoma, breast cancer, cancers of the brain and central nervous system, and cervical cancer) and among men (testicular cancer, malignant melanoma, cancers of the brain and central nervous system, and Hodgkin's lymphoma) separately.

## Methods

### The Danish life course (DANLIFE) cohort

**Fig. 1:**
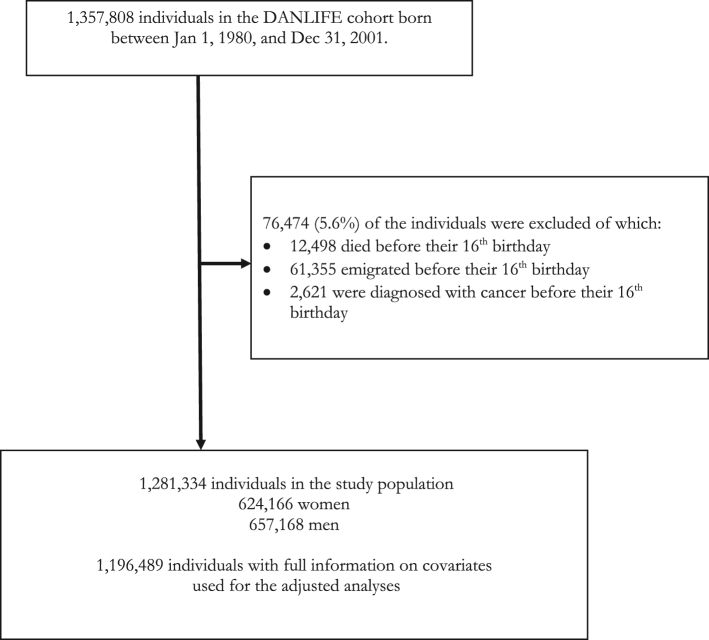
Flow chart of the study population.

We used data from a large register-based life course cohort (DANLIFE) based on comprehensive information from the Danish nationwide registers.^18^ Every Danish citizen is given a unique personal identification number at birth, which permits exact linkage between information from administrative and research registries in Denmark. Access to Danish administrative and health registers is granted by *Statistics Denmark* and *the Danish Health Data Authorities*. The DANLIFE cohort includes all children born in Denmark from 1980 and onwards. Those who immigrate to Denmark after birth are not included. To ensure adversity data on entire childhoods (from 0 to 15 years), we restricted the sample to 1,281,334 individuals who were born between 1980 and 2001, and who were alive, had not emigrated, and had not been diagnosed with cancer before their 16th birthday (Fig. 1). For the adjusted analyses, we restricted the sample to 1,196,489 with full information on all covariates (maternal and paternal age at birth, parental country of origin, parental history of cancer, being born preterm, being born small for gestational age, birth year). The study population was followed from their 16th birthday until emigration, death, or the end of follow-up on 31 Dec 2018. Individuals who emigrated (n = 98,152) or died (n = 5255) during follow-up were censored at the date of emigration/death.

### Childhood adversity

The linkage between child, parents, and siblings in the Danish registers enables the assessment of various types of childhood adversity. We accounted for the complexity and time-varying nature of childhood adversity by integrating information on the duration and timing of childhood adversity across multiple dimensions based on annually updated data extracted from the registers. A panel of experts in stress, child health, and child psychology have previously decided on three predefined dimensions of childhood adversity based on theoretical insights: a) material deprivation (i.e., family poverty and parental long-term unemployment); b) loss or threat of loss within the family (i.e., parental severe somatic illness, sibling severe somatic illness, and death of a parent or a sibling); and c) family dynamics (i.e., maternal separation, being placed in foster care, parental psychiatric illness, sibling psychiatric illness, and parental alcohol or drug abuse).^16^ Supplementary Table S1 provides an overview of the included childhood adversity measures.

We have previously used the Stata package TRAJ to fit trajectory clusters using a zero-inflated Poisson likelihood with a cubic trajectory functions. This model estimated a probability for each individual of being part of a given trajectory group,^19^ and we visually judged that 5 trajectory groups divided the individuals optimally^16^ (see the supplementary method section for details about the analytical approach). Supplementary Fig S1 shows the five modelled trajectory groups. In this sample, 693,584 (54%) of the children belonged to the *Low Adversity* group, which was characterized by a consistent low rate of adversities across all three dimensions. The *Early Life Material Deprivation* trajectory group (257,340 children, 20%) was characterized by a high annual rate of material deprivation during the first years of life after which the rate became very low. The *Persistent Material Deprivation* trajectory group (171,321 children, 13%) was characterized by a high annual rate of material deprivation during the entire childhood, but with a low rate of adversities in the other two dimensions. The *Loss or Threat of Loss* trajectory group (117,675 children, 9%) was characterised by a relatively high and increasing annual rate of loss or threat of loss during childhood. The *High Adversity* trajectory (41,414 children, 3%) was characterized by a high and increasing annual rate of adversities across all three dimensions.

### Cancer outcomes

We utilized data from the Danish Cancer Registry and the Danish Register for Causes of Death to identify three types of cancer outcomes: i) first-time cancer incidence defined as first cancer diagnosis, ii) cancer mortality rate defined as death due to cancer, and iii) 5-year case fatality rate defined as any cause of death within five years of primary cancer diagnosis. Cancer was defined as cancer of all sites excluding non-melanoma skin cancer (C44) in accordance with the classifications used by the Association of the Nordic Cancer registries (www.nordcan.iarc.fr/en) using the following ICD-10 codes: C00–C43, C45–C99, D09.0–D09.1, D30.1–D30.9 D32–D33, D35.2–D35.4, D41.0–D41.9, D42–D43, D44.3–D44.5, D45–D47. For cancer incidence, we also assessed the four most common types of cancer among women (malignant melanoma, C43; breast cancer, C50; cancers of the brain and central nervous system (CNS), C70–C72, C75.1–C75.3, D32–D33, D35.2–D35.4, D42–D43, D44.3–D44.5; cervical cancer, C53)) and men (testicular cancer, C62; malignant melanoma; cancers of the brain and central nervous system; Hodgkin's lymphoma, C81). Mortality is very low in this age group, so we were unable to assess cancer mortality and case-fatality for subtypes of cancer.

### Statistical analysis

Exploratory data analysis involved fitting smoothed cumulative risk curves using Kaplan Meier estimators for overall cancer, and separately for the subtypes of cancer among women and men (we smoothed the curves for data protection reasons). We subsequently estimated hazard ratios (HR) and 95% CIs for the three different outcomes in a Cox proportional hazards model with trajectories of childhood adversity as the exposure variable in a subpopulation with full information on relevant covariates. The proportional hazards assumption was valid. Age was used as the underlying timescale for analysis on cancer incidence and mortality, and time since diagnosis was used as the underlying timescale for case fatality. We also estimated hazard differences using the Aalen's additive hazard model to assess the absolute cancer burden associated with each trajectory group. The relation between the cumulative risk curves over time was assessed visually and judged to be reasonably stable, which supported reporting average hazard ratios and hazard differences during follow-up.^20^ All analyses accounted for siblings sharing the same environment (clustered by mother's IDs). The estimates were adjusted for maternal and paternal age at birth, parental country of origin, parental history of cancer, being born preterm, being born small for gestational age, birth year and additionally parental educational level in supplementary analyses. The underlying cancer types are different for men and women and all analyses were therefore stratified by sex. In a sensitivity analysis, the population was restricted to those without parental history of cancer to prevent confounding from genetic predisposition. Data were prepared using Stata and survival analyses were conducted in R (the package ‘survival’ was used for the Cox model and the package ‘timereg’ was used for the Aalen model).

### Ethical issues

The DANLIFE study has been approved by the Danish Data Protection Agency through the records of research projects (involving personal data) at the Faculty of Health and Medical Sciences, University of Copenhagen (record no 514-0641/21-3000). The Danish Data Protection Agency ensures compliance with national and EU legislation. Registry linkage studies do not require ethical approval by the Danish National Committee on Health Research Ethics according to Danish Law.

### Role of funding sources

The funders had no role in the study design; in the collection, analysis, and interpretation of data; in the writing of the paper or the decision to submit the paper.

## Results

### Cancer burden in adolescents and young adults

**Fig. 2:**
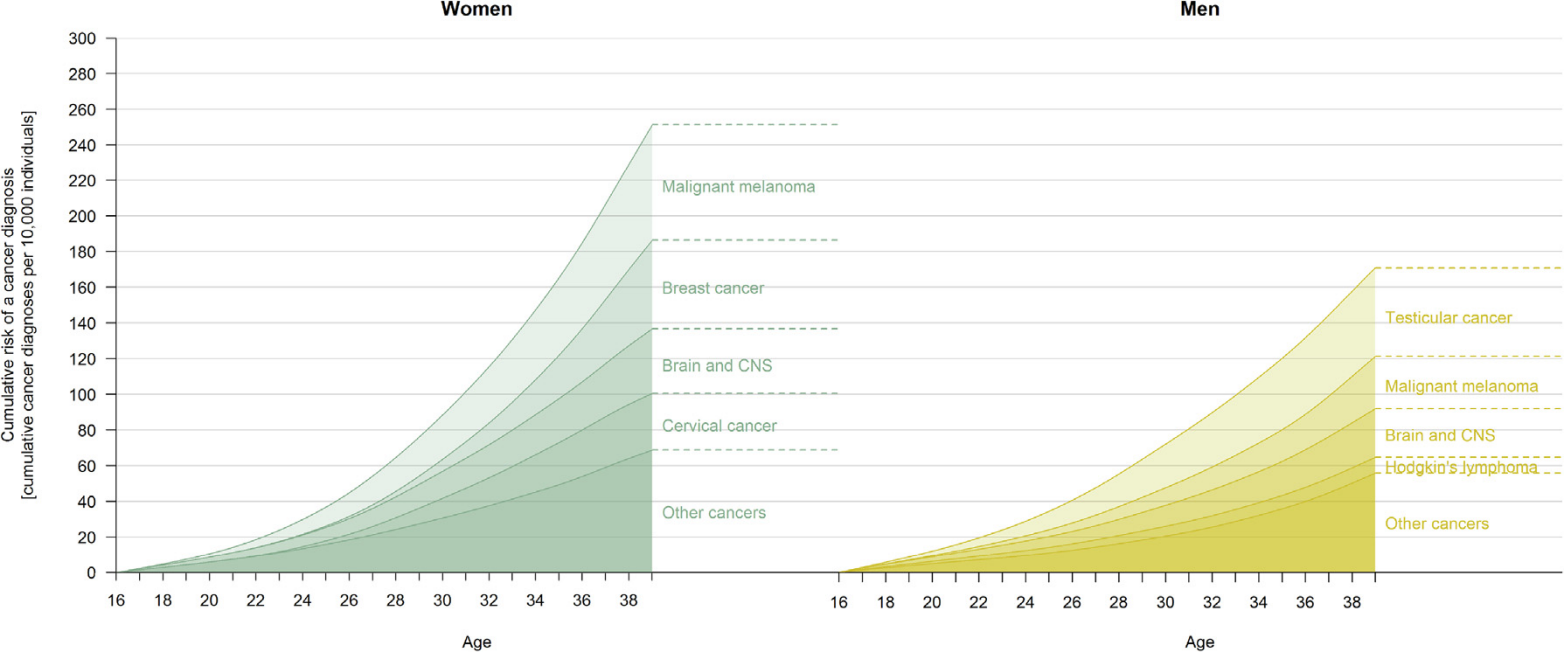
Cumulative incidence of overall cancer and site-specific cancers among the full sample of 1,281,334 young men and women (aged 16–38 years).

The dataset covered 624,166 women and 657,168 men followed prospectively from birth with cancer outcome data from age 16 and up to a maximum age of 38 years. The median follow-up time from age 16 years was 10.1 years, ranging from 1 day to 23 years. During follow-up, incident cancers occurred among 4470 women and 3759 men and cancer deaths among 330 women and 332 men. Fig. 2 shows the cumulative incidence patterns of overall and site-specific cancer in men and women.

### Childhood adversity and overall cancer burden

**Fig. 3:**
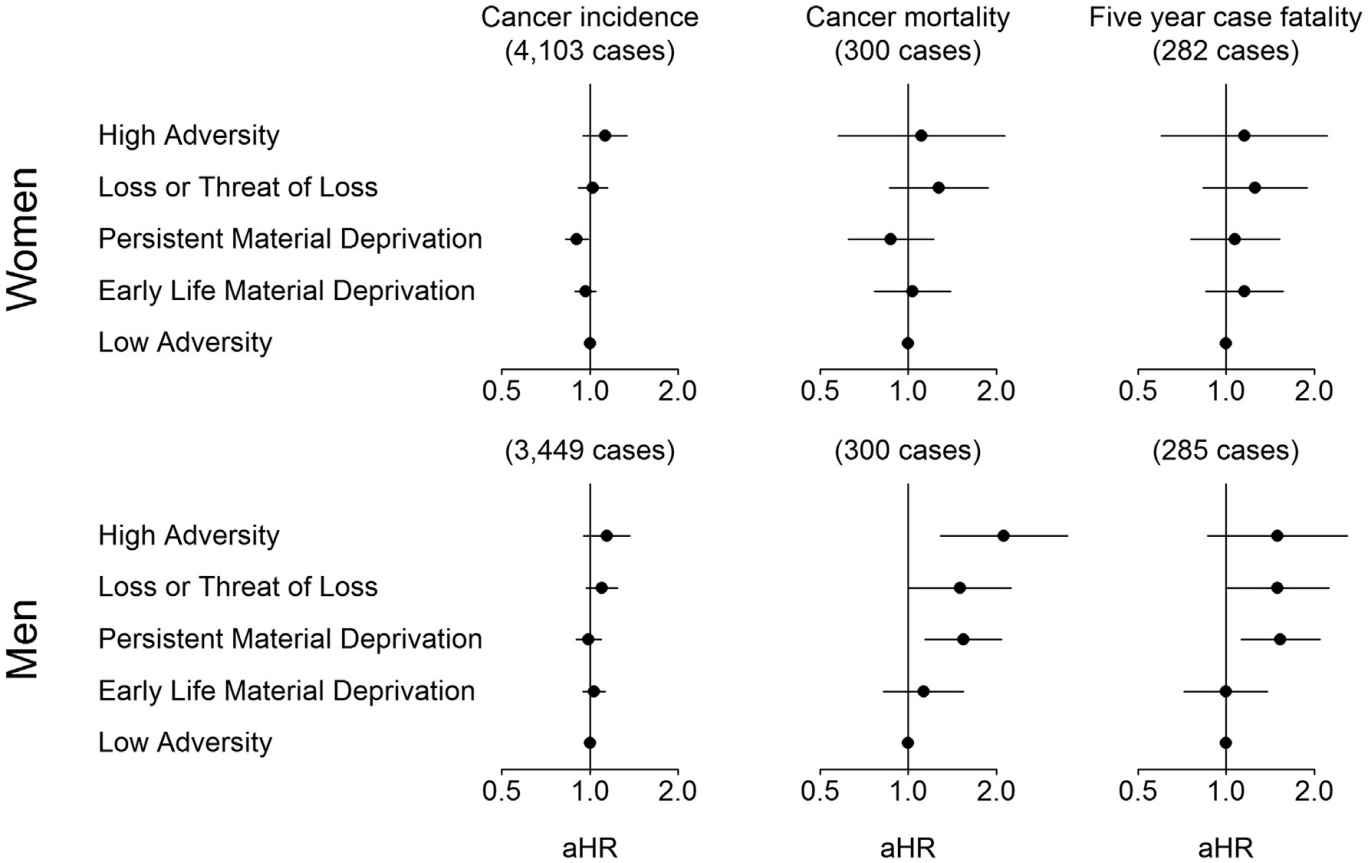
Adjusted hazard ratios (aHR) and 95% CI for cancer incidence, cancer mortality and 5-year case-fatality in 1,196,489 adolescents and young adults (16–38 years) according to trajectory groups of childhood adversity. Adjusted for parental age at birth, parental country of origin, parental history of cancer, being born preterm, being born small for gestational age, and birth year.

Childhood adversity was associated with parental country of origin, young parental age at birth, being born preterm, and being small for gestational age (Table 1). As expected, a larger proportion of those in the *Loss or Threat of Loss* group had a parental history of cancer compared to the other groups. Fig. 3 summarises the adjusted associations between the trajectory groups of childhood adversity and overall cancer incidence, cancer mortality and 5-year case fatality. For women, the persistent material deprivation group (HR 0.90; 95% CI 0.82; 0.99) had a slightly lower incidence rate of cancer than the low adversity group, whilst the incidence rate in the other groups was comparable to that in the low adversity group. Also, there were no consistent associations between childhood adversity and overall cancer mortality or case-fatality in women.

**Table 1:** Background characteristics at time of birth according to the five estimated trajectory groups.

	Low adversity (693,584)	Early life material deprivation (257,340)	Persistent material deprivation (171,321)	Loss or threat of loss (117,675)	High adversity (41,414)
Sex, %					
Men	51	51	51	51	54
Women	49	49	49	49	46
Parental origin, %					
Non-European	≤1	4	8	3	2
European	99	96	92	97	98
Missing	≤1	≤1	≤1	≤1	≤1
Maternal age, %					
<20 years	≤1	4	7	3	11
20–30 years	65	73	71	63	68
>30 years	34	24	22	33	22
Missing	≤1	≤1	≤1	≤1	≤1
Paternal age, %					
<20 years	≤1	≤1	≤1	≤1	2
20–30 years	46	55	54	45	50
>30 years	52	40	40	51	37
Missing	2	4	4	3	10
Preterm birth, %					
No	92	92	91	90	88
Yes	5	5	5	7	9
Missing	3	3	4	3	4
Small for gestational age, %					
No	85	83	80	82	74
Yes	11	14	16	15	22
Missing	3	3	4	3	4
Parental history of cancer, %					
No	80	82	79	66	81
Yes	20	18	21	34	19
Parental education, %					
<10 years	8	21	32	21	54
10–12 years	48	55	49	50	36
≥12 years	44	23	18	29	10
Missing	≤1	≤1	≤1	≤1	≤1

Parental origin (European descent [Europe, North America, Australia and New Zealand], non-European descent if both parents are of non-European descent), maternal age at time of birth (<20 years, 20–30 years, >30 years), being born preterm (before completion of week 37 of gestation), being born small for gestational age (defined as birthweight below the 10th percentile of sex-specific standard reference curves for intrauterine growth) and parental history of cancer (mother or father has a cancer diagnosis), parental education (highest attained education level by either the mother or the father).

For men, there was no clear association between childhood adversity and incidence of cancer, but an association was found between childhood adversity and both cancer mortality and case fatality. Compared with that of the low adversity group, the cancer mortality rate was higher in the persistent material deprivation group (HR 1.72; 95% CI 1.29; 2.31), the loss and threat of loss group (HR 1.56; 95% CI 1.05; 2.32) and the high adversity group (HR 2.27 95% CI 1.38; 3.72). In absolute numbers, the latter estimate corresponds to 4.3 additional cancer deaths (95% CI 0.4; 8.2) among 100,000 men in this age group per year (see Supplementary Table S2 for all risk estimates) Similar associations were found for case fatality in men. Further adjustment for parental education had very little impact on the results (Supplementary Table S3). Restricting the analyses to a subpopulation without a parental history of cancer attenuated the risk estimates somewhat, but the association between high adversity and cancer mortality remained unchanged (HR 2.27; 95% CI 1.28; 4.02) (Supplementary Fig. S4).

### Subtypes of cancer

Table 2 summarises the adjusted associations between childhood adversity and the incidence of subtypes of cancer. The unadjusted estimates are shown in Supplementary Table S5. The four most common subtypes of cancer among women in this age-group were malignant melanoma (1096 cases), breast cancer (465 cases), cancers of the brain and CNS (689 cases), and cervical cancer (470 cases). Compared with the low adversity group, women in all other groups had a lower incidence rate of malignant melanoma. The strongest associations were found for the persistent material deprivation group, where 6.3 fewer cases of malignant melanoma (95% CI: 3.2; 9.3) were diagnosed annually per 100,000 women compared to the low adversity group. Women in this group also had a lower risk of brain and CNS cancer (HR 0.74; 95% CI 0.58; 0.94) but a higher risk of breast cancer (HR 1.27; 95% CI 1.00; 1.62) compared to the low adversity group. However, these associations attenuated and became statistically non-significant in a sensitivity analysis limited to women without a parental history of cancer (Supplementary Table S6). Women in the high vs low adversity group had a higher risk of breast cancer (HR 1.71; 95% CI 1.09; 2.70) and cervical cancer (HR 1.82; 95% CI 1.18; 2.83). In a subpopulation without parental cancer, the risk of cervical cancer was attenuated (HR 1.51; 95% CI 0.87; 2.64), while the risk of breast cancer became slightly stronger (HR 1.99; 95% CI 1.18; 3.36).

**Table 2:** Adjusted hazard ratio (aHR) and adjusted hazard difference (aHD) per 100,000 individuals per year for cancer incidence according to childhood adversity trajectories among 582,595 young women and 613,894 young men.

Women												
	Malignant melanoma (n = 1096)			Breast cancer (n = 465)			Brain and CNS (n = 689)			Cervical cancer (n = 470)		
	No cases	aHR (95% CI)	aHD (95% CI)	No cases	aHR (95% CI)	aHD (95% CI)	No cases	aHR (95% CI)	aHD (95% CI)	No cases	aHR (95% CI)	aHD (95% CI)
Low adversity	620	1 (ref)	0 (ref)	209	1 (ref)	0 (ref)	355	1 (ref)	0 (ref)	204	1 (ref)	0 (ref)
Early life deprivation	212	0.81 (0.69; 0.95)	−3.7 (−6.4; −1.0)	90	1.05 (0.82; 1.35)	0.4 (−1.3; 2.1)	158	1.06 (0.87; 1.28)	0.7 (−1.6; 3.0)	105	1.18 (0.93; 1.50)	1.2 (−0.6; 3.0)
Persistent deprivation	148	0.71 (0.59; 0.85)	−6.3 (−9.3; −3.2)	103	1.27 (1.00; 1.62)	2.1 (−0.2; 4.4)	84	0.74 (0.58; 0.94)	−3.0 (−5.3; −0.8)	96	1.23 (0.96; 1.57)	1.7 (−0.6; 3.9)
Loss/threat of loss	86	0.86 (0.68; 1.07)	−3.0 (−6.8; 0.9)	42	1.25 (0.90; 1.74)	1.5 (−1.1; 4.1)	67	1.11 (0.85; 1.44)	1.3 (−2.0; 4.6)	42	1.30 (0.93; 1.82)	1.9 (−0.7; 4.4)
High adversity	30	0.83 (0.58; 1.21)	−3.3 (−9.5; 2.9)	21	1.71 (1.09; 2.70)	4.8 (−0.3; 9.9)	25	1.16 (0.77; 1.75)	2.0 (−3.6; 7.5)	23	1.82 (1.18; 2.83)	5.7 (0.4; 11.0)

**Men**												
	Testicular cancer (n = 1079)			Malignant melanoma (n = 481)			Brain and CNS (n = 569)			Hodgkin's lymphoma (n = 255)		
	No cases	aHR (95% CI)	aHD (95% CI)	No cases	aHR (95% CI)	aHD (95% CI)	No cases	aHR (95% CI)	aHD (95% CI)	No cases	aHR (95% CI)	aHD (95% CI)
Low adversity	542	1 (ref)	0 (ref)	270	1 (ref)	0 (ref)	274	1 (ref)	0 (ref)	128	1 (ref)	0 (ref)
Early life deprivation	240	1.07 (0.92; 1.25)	1.2 (−1.5; 3.8)	91	0.81 (0.64; 1.03)	−1.6 (−3.2; 0.1)	127	1.14 (0.92; 1.41)	1.1 (−0.7; 3.0)	55	1.07 (0.78; 1.48)	0.3 (−1.0; 1.5)
Persistent deprivation	177	1.01 (0.85; 1.19)	0.0 (−2.9; 3.0)	68	0.75 (0.57; 0.98)	−2.3 (−4.2; −0.4)	95	1.15 (0.91; 1.47)	1.3 (−0.9; 3.4)	35	0.91 (0.62; 1.35)	−0.3 (−1.7; 1.0)
Loss/threat of loss	80	0.93 (0.73; 1.17)	−1.2 (−4.7; 2.3)	38	0.88 (0.62; 1.24)	−1.1 (−3.4; 1.4)	55	1.26 (0.93; 1.70)	2.1 (−0.8; 4.9)	29	1.35 (0.90; 2.03)	1.4 (−0.7; 3.4)
High adversity	40	1.15 (0.83; 1.59)	2.4 (−3.6; 8.4)	14	0.82 (0.47; 1.41)	−1.5 (−5.1; 2.1)	18	1.06 (0.66; 1.71)	0.5 (−3.5; 4.5)	8	1.03 (0.50; 2.11)	0.1 (−2.6; 2.8)

a: Adjusted for parental age at birth, parental country of origin, parental history of cancer, being born preterm, being born small for gestational age, and birth year.

The four most common subtypes of cancer among men in this age group were testicular cancer (1079 cases), malignant melanoma (481 cases), cancers of the brain and CNS (569 cases), and Hodgkin's lymphoma (255 cases). Similar to women, childhood adversity was associated with a slightly lower incidence of malignant melanoma among men, particularly for the persistent material deprivation group compared to the low adversity group (HR 0.75; 95% CI 0.57; 0.98). An association which attenuated in a sensitivity analysis restricted to men without parental cancer (HR 0.79; 95% CI 0.57; 1.11) (Supplementary Table S6) Apart from this, there were no clear associations between childhood adversity and incidence of testicular cancer, brain and CNS cancer, or Hodgkin's lymphoma. Adjustment for covariates only had a minor impact on the results (Supplementary Table S5).

## Discussion

Based on unselected life-course data from a population of 1.2 million men and women, we show a considerable cancer burden among adolescents and young adults. Cancer is a heterogeneous group of diseases with different underlying etiology, and we hypothesized that childhood adversity would affect specific subtypes of cancer differently. In support of this hypothesis, we found that young women who had experienced persistent material deprivation during childhood carried a slightly lower risk of overall cancer incidence, particularly due to malignant melanoma and brain and CNS cancers, and a slightly higher risk of breast cancer. These associations seemed to be partly explained by parental history of cancer. Further, women who had been exposed to childhood adversity across various dimensions seemed to carry a moderately higher risk of breast and cervical cancer than those with low adversity. For men, there were no clear associations between childhood adversity and overall cancer, Hodgkin's lymphoma, testicular, brain and CNS cancers. We also hypothesized that childhood adversity may impact cancer survival though socially patterned differences in biological susceptibility, access to support and care, and treatment adherence. Here, we found that men who had experienced adversity during childhood, especially the persistent material deprivation and high adversity groups, seemed to carry a disproportionate burden of cancer mortality and case fatality during adolescence or young adulthood compared to their peers.

Previous studies on childhood adversity and cancer have focused on cancer incidence, primarily, amongst older adults. Some of these studies have found a higher risk of cancer incidence associated with childhood adversity, while others have not.^10^^,^^11^ Overall, we find no indication of a higher risk of overall cancer incidence associated with accumulation of high adversity during childhood. The underlying pattern is however more diverse, with childhood adversity being associated with a lower risk of some subtypes of cancer (malignant melanoma and brain and CNS cancer) and a higher risk of others (breast and cervical cancer), particular for women. This emphasizes the importance of not studying cancer as a single disease. Underlying mechanisms may involve differential exposure to environmental and behavioral risk factors such as sun exposure, smoking, alcohol intake and air pollution, co-morbidities such as overweight or infections, combined with barriers to access to health services and care including HPV vaccination schedules and cancer screening. These mechanisms remain speculative and warrant further investigation to identify entry points for intervention.

Adolescents and young adults with cancer are an understudied group with special needs for treatment and care, making it important to identify vulnerable subgroups who should be specifically targeted to ensure that their treatment and information needs are met. We show a higher cancer mortality and case fatality rate in young men who had been exposed to persistent material deprivation and/or accumulation of adversity during childhood. Facing a cancer diagnosis and navigating the various transitions and treatments of the cancer journey is challenging and complex,^3^ and many cancer patients rely on a strong supportive network often provided by parents and close family to manage the situation. However, children who grow up in families with a high degree of adversity, e.g., psychiatric illness, abuse, or violence in the family, may lack access to such support. Furthermore, we have previously documented a massive disease burden among children and young adults who have experienced childhood adversity,^17^ and cancer patients within these groups are therefore likely to suffer from other co-morbidities in addition to cancer, including mental health problems, which may render their treatment more complex and potentially affect treatment seeking and adherence behaviors. The sex difference is puzzling, but previous studies have for example found more unmet information needs related to cancer among young men than women.^21^ Sex differences in treatment seeking behavior and treatment adherence may also add to these observed differences.

Other mechanisms underlying a higher case fatality among young men with cancer remain speculative but may involve both biological and social processes. Converging evidence from in vitro, in-vivo and clinical studies shows that stress-related processes are linked to elements of tumor progression in both animal and human models.^22^ Both stress-induced adrenergic pathways and elevated levels of glucocorticoids appear to be directly involved in tumor growth and progression. The underlying mechanisms include effects on the cellular immune response, angiogenesis, invasion, anoikic, and inflammation. Individuals with adolescent and young onset cancer may also face financial and social challenges that may delay access to appropriate care, timely diagnosis, and treatment.^23^ There is no specific consensus or guidelines on the treatment of this age group, and barriers to appropriate treatment include lower availability and more concerns related to involvement with clinical trials, financial issues, and lack of supportive care focused on the special needs of this group.^21^ These mechanisms may further be amplified by social vulnerability. The higher case fatality rate observed among young socially disadvantaged men is particularly concerning since this finding is nested within a country with a strong social security system and universal and free health care, and indicates that there are other aspects involved, such as delay in diagnosis, appropriate treatment, and adherence to treatment.

Our study has strengths and limitations. The findings are based on information on childhood adversity from nationwide registers measured prospectively throughout the entire childhood in a nationwide unselected population, and the large sample size allowed for analyses across cancer subtypes. Relying on registry-linkage data ensured complete long-term prospective follow-up and prevented problems with selective inclusion. It also allowed us to account not only for adversities from different domains of life, but also for their accumulation throughout the life course. However, only a limited number of indicators of childhood adversity were available in the registers. These adversities cover dimensions of material deprivation, loss or threat of loss and family dynamics, but do not directly measure e.g., violence, sexual abuse, or neglect in the families or bullying in schools, which have previously been found to be related to various health outcomes.^10^ Also, we derived information on alcohol abuse from hospitalizations and medication use related to alcohol abuse, but most cases of alcohol abuse are never registered. While this is a limitation, we specifically included measures on foster care, which is closely related to known cases of violence, abuse, and neglect in the family. It is also well-known that adversities tend to cluster, and by including a broad range of adversity measures we expect to have been able to capture at least the most severe cases of childhood adversity, but we are likely to have underestimated the true effect of childhood adversity to some degree. Also, the cohort was restricted to children born in Denmark, and the results can therefore not be generalized to e.g., children who experience adversity related to migration or war.

The age range used to define adolescent and young adult cancers is flexible, but most often aligns with the age range 15–39 years proposed by the Progress Review Group.^24^ We started follow-up at age 16 to ensure temporality between childhood adversity (0–15 years) and cancer diagnosis. Only few incident cancer cases (n = 245) occurred between 15 and 16 years of age, and we do not expect the 1-year delay in follow-up to have impacted our overall findings. Furthermore, we specifically focused on cancers among adolescents and young adults and the findings cannot be directly generalized to older populations with a different cancer burden. Finally, we adjusted for parental country of origin as a crude proxy for ethnicity, but we did not have actual information on ethnicity or race, which may play a role in cancers such as melanoma. However, ethnic minority populations in Denmark make up only a small proportion of the population (<10%).

We extend the existing literature by not only looking at cancer incidence, but also include cancer mortality and case-fatality. Still, this does not capture the full cancer burden, and socially vulnerable individuals may be more likely to present with more advanced stages of disease or have repeated relapses that may be curable but involve intense treatment with a high burden of late effects. Our study indicates a higher mortality burden associated with childhood adversity and future research should look more into these other important indicators of the cancer burden. Further, case fatality rates are low in young adulthood, and we were therefore not able to look at case fatality for specific subtypes of cancer. Larger studies are needed to explore these findings further.

In conclusion, we found childhood adversity to be differentially associated with different subtypes of cancer in adolescents and young adults. In particular, we found a higher risk of breast and cervical cancer incidence among young women exposed to childhood adversity, which needs further exploration. We also found that men who had experienced social adversity during childhood carried a disproportionate burden of cancer mortality and case fatality during adolescence or young adulthood compared to their peers. This is a formative period of life and a cancer diagnosis during these years can have a major impact on life trajectories. Previous studies have shown a substantial impact of cancer during adolescence and young adulthood on work, educational attainment, and family formation.^21^ Exposure to persistent adversity during childhood may create a social vulnerability which amplifies these effects and makes some subgroups particularly vulnerable to the long-term social, economic and health consequences of cancer in young age. This calls for appropriate treatment and support specifically aimed at young and socially vulnerable cancer patients in clinical practice.

## Contributors

NHR, JB, LKE, MD, DT-R, SB and AR conceived the idea and designed the study. JB did the data linkage and data cleaning for DANLIFE. AR and SB did the statistical analyses. NHR, JB, SB and AR had access to raw data due to data protection rules, but all authors had access to summary data. NHR wrote the first draft of the manuscript. All authors discussed the results, contributed to the final manuscript, and had final responsibility for the decision to submit for publication.

## Data sharing statement

The data material contains personally identifiable and sensitive information. According to the Act on Processing of Personal Data, such data cannot be made publicly available. Inquiries about secure access to data under conditions stipulated by the Danish Data Protection Agency should be directed to the corresponding author.

## Declaration of interests

JB is currently an employee at Novo Nordisk A/S and AR is currently an employee at Lundbeck A/S. They contributed to this manuscript during their previous academic positions at the University of Copenhagen, and not during their current positions. The other authors report no conflicts of interest.

## Appendix A Supplementary data

Supplementary material R2

ABSTRACT
