# Supplementary material for: Cancer burden among adolescents and young adults in relation to childhood adversity: a nationwide life-course cohort study of 1.2 million individuals
Source: Lancet Reg Health Eur. 2023 Feb 10;27:100588. doi: 10.1016/j.lanepe.2023.100588 (PMC9945708; doi:10.1016/j.lanepe.2023.100588)
Supplement: Supplementary material R2 [file mmc1.pdf]

SUPPLEMENTARY MATERIAL

Overview

Supplementary Table 1. Dimensions and definitions of childhood adversities in the five trajectory groups of adversity.

Supplementary Figure 1. Trajectories of childhood adversities across predefined dimensions of material deprivation, loss or threat of loss, and family dynamics presented as rates per person-year (PYR).

Supplementary Table 2. Hazard ratios (HR), hazard difference (HD) per 100,000 individuals per year and 95% confidence intervals (CI) for cancer incidence, cancer mortality and 5-year case-fatality among 1,194,316 individuals with full covariate information according to trajectory groups of childhood adversity.

Supplementary Table 3. Sensitivity analysis adjusting further for parental education. Hazard ratios (HR), hazard difference (HD) per 100,000 individuals per year and 95% confidence intervals (CI) for cancer incidence, cancer mortality and 5-year case-fatality among 1,194,316 individuals with full covariate information according to trajectory groups of childhood adversity.

Supplementary Figure 4. Sensitivity analysis restricted to a subpopulation without parental history of cancer. Adjusted hazard ratios (aHR) and 95% CI for cancer incidence, cancer mortality and 5-year case-fatality.

Supplementary Table 5. Unadjusted hazard ratio (HR) and hazard difference (HD) per 100,000 individuals per year for cancer incidence according to childhood adversity trajectories among 582,595 young women and 613,894 young men.

Supplementary Table 6. Sensitivity analyses restricted to a population without family history of cancer. Adjusted hazard ratio (aHR) and adjusted hazard difference (aHD) per 100,000 individuals per year for cancer incidence according to childhood adversity trajectories among 459,546 young women and 484,900 young men.

Supplementary Methods. Group-based multi-trajectory model.

Supplementary Table 1. Dimensions and definitions of childhood adversities in the five trajectory groups of adversity identified by Rod et al. (1). Details described in Bengtsson et al. (2). The authors on Rod et al. (1) included experts in stress, child health, and child psychology and this panel of experts decided on the three predefined dimensions of childhood adversity.

	Adversity	Definition	Registers
**Material deprivation**	**Family poverty**	Family income below 50% of the median national family income in a given year	The Income Statistics Register (3)
	**Parental long-term unemployment**	A parent being unemployed for at least 12 months	The Integrated Database for Labour Market Research (4)
**Loss or threat of loss**	**Death of a parent**	Death of a parent	The Danish Civil Registration System (5)
	**Death of a sibling**	Death of a sibling	The Danish Civil Registration System (5)
	**Parental somatic illness**	A parent being diagnosed with one of the diseases included in the Charlson comorbidity index	The Danish National Patient Register (6)
	**Sibling somatic illness**	A sibling being diagnosed with one of the seven somatic illnesses most commonly related to mortality in children aged 0–18 years in Denmark: malignant neoplasm; congenital anomalies of the heart and circulatory system; congenital anomalies of the nervous system; cerebral palsy; epilepsy; cardiomyopathy; congenital disorders of lipid metabolism	The Danish National Patient Register (6)
**Family dynamics**	**Foster care**	Being placed in out-of-home care	The Register of Support for Children and Adolescents (7)
	**Parental psychiatric illness**	A parent being admitted for at least 1 day to a psychiatric hospital or ward with a primary diagnosis related to psychiatric illness (excluding primary diagnoses related to alcohol and drug abuse)	The Danish Psychiatric Central Research Register (8); The Danish National Patient Register (6)
	**Sibling psychiatric illness**	A sibling being admitted for at least 1 day to a psychiatric hospital or ward with a primary diagnosis related to psychiatric illness	The Danish Psychiatric Central Research Register (8); The Danish National Patient Register (6)
	**Parental alcohol abuse**	A parent being diagnosed with a disease related to alcohol abuse or buying a prescribed drug used in treatment of alcohol dependence	The Danish Psychiatric Central Research Register (8); The Danish National Patient Register (6); The Danish National Prescription Registry (9)
	**Parental drug abuse**	A parent being diagnosed with a disease related to drug abuse or buying a prescribed drug used in treatment of drug dependence	The Danish Psychiatric Central Research Register (8); The Danish National Patient Register (6); The Danish National Prescription Registry (9)
	**Maternal separation**	The mother no longer sharing address with a partner	The Danish Civil Registration System (5)

References to Supplementary Tabl2 1

1. Rod NH, Bengtsson J, Budtz-Jørgensen E, Clipet-Jensen C, Taylor-Robinson D, Andersen A-MN, et al. Trajectories of childhood adversity and mortality in early adulthood: a population-based cohort study. The Lancet. 2020 Aug 15;396(10249):489–97.

2. Bengtsson J, Dich N, Rieckmann A, Rod NH. Cohort profile: the DANish LIFE course (DANLIFE) cohort, a prospective register-based cohort of all children born in Denmark since 1980. BMJ Open. 2019 Sep 20;9(9):e027217.
3. Baadsgaard M, Quitzau J. Danish registers on personal income and transfer payments. Scand J Public Health. 2011;39(7 Suppl):103-105.
4. Petersson F, Baadsgaard M, Thygesen L. Danish registers on personal labour market affiliation. Scand J Public Health. 2011;39(7 Suppl):95-98.
5. Schmidt M, Pedersen L, Sørensen HT. The Danish Civil Registration System as a tool in epidemiology. Eur J Epidemiol. 2014;29(8):541-549. doi:10.1007/s10654-014-9930-3.
6. Schmidt M, Schmidt SAJ, Sandegaard JL, Ehrenstein V, Pedersen L, Sørensen HT. The Danish National Patient Registry: A review of content, data quality, and research potential. Clin Epidemiol. 2015;7:449-490. doi:10.2147/CLEP.S91125.
7. Description of the Register of Support for Children and Adolescents [in Danish]. https://www.dst.dk/da/TilSalg/Forskningsservice/Dokumentation/hoejkvalitetsvariable/stoette-til-udsatte-boern-og-unge. Accessed on June 26 2022.
8. Mors O, Perto G, Mortensen P. The Danish Psychiatric Central Research Register. Scand J Public Health. 2011;39(7 Suppl):54-57.
9. Pottegård A, Schmidt SAJ, Wallach-Kildemoes H, Sørensen HT, Hallas J, Schmidt M. Data resource profile: The Danish National Prescription Registry. Int J Epidemiol. 2017;46(3):798. doi:10.1093/ije/dyw213

Supplementary Figure 1. Trajectories of childhood adversities across predefined dimensions of material deprivation, loss or threat of loss, and family dynamics presented as rates per person-year (PYR). The percentages refer to the proportion of children belonging to each trajectory group in the sample, e.g., 3% of the children belong to the high adversity group.

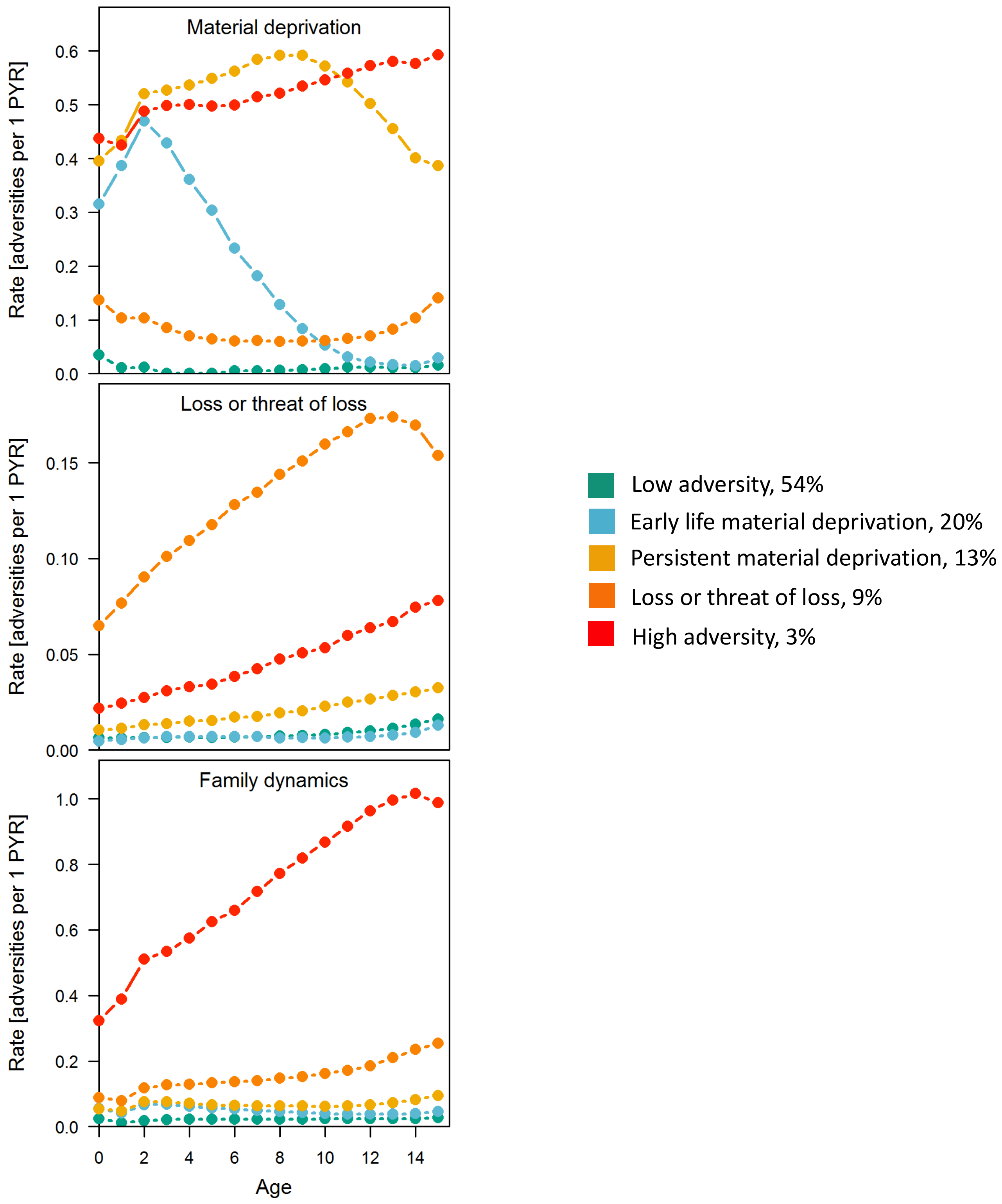

Supplementary Table 2. Hazard ratios (HR), hazard difference (HD) per 100,000 individuals per year and 95% confidence intervals (CI) for cancer incidence, cancer mortality and 5-year case-fatality among 1,196,489 individuals with full covariate information according to trajectory groups of childhood adversity.

	**Men** (n=613,894)				**Women** (n=582,595)			
	**Cancer Incidence** First time hospitalization with a cancer							
	No. of incident cases: 3449				No. of incident cases: 4103			
	HR (95% CI)	aHR (95% CI)	HD (95% CI)	aHD (95% CI)	HR (95% CI)	aHR (95% CI)	HD (95% CI)	aHD (95% CI
Low Adversity	1 (ref)	1 (ref)	0 (ref)	0 (ref)	1 (ref)	1 (ref)	0 (ref)	0 (ref)
Early Life Deprivation	1.03 (0.94; 1.12)	1.03 (0.95; 1.13)	1.5 (-3.1; 6.0)	1.7 (-2.9; 6.3)	0.97 (0.89; 1.05)	0.97 (0.89; 1.05)	-2.1 (-7.4; 3.1)	-2.3 (-7.7; 3.0)
Persistent Deprivation	0.96 (0.87; 1.06)	0.99 (0.90; 1.09)	-2.2 (-7.3; 2.9)	-0.7 (-6.0; 4.5)	0.88 (0.80; 0.96)	0.90 (0.82; 0.99)	-9.1 (-15.0; -3.3)	-7.3 (-13.4; -1.2)
Loss/Threat of Loss	1.12 (0.99; 1.26)	1.10 (0.97; 1.24)	5.9 (-0.8; 12.6)	4.7 (-2.0; 11.5)	1.06 (0.94; 1.18)	1.02 (0.91; 1.15)	3.7 (-3.9; 11.3)	1.2 (-6.4; 8.9)
High Adversity	1.12 (0.94; 1.35)	1.14 (0.95; 1.37)	6.4 (-4.1; 16.9)	7.2 (-3.3; 17.7)	1.12 (0.94; 1.33)	1.13 (0.95; 1.34)	8.4 (-4.7; 21.5)	8.6 (-4.7; 21.8)
	**Cancer Mortality** Death due to cancer							
	No. of cancer deaths: 300				No. of cancer deaths: 300			
	HR (95% CI)	aHR (95% CI)	HD (95% CI)	aHD (95% CI	HR (95% CI)	aHR (95% CI)	HD (95% CI)	aHD (95% CI
Low Adversity	1 (ref)	1 (ref)	0 (ref)	0 (ref)	1 (ref)	1 (ref)	0 (ref)	0 (ref)
Early Life Deprivation	1.17 (0.86; 1.59)	1.13 (0.82; 1.55)	0.6 (-0.7; 1.9)	0.5 (-0.8; 1.8)	1.06 (0.79; 1.42)	1.04 (0.77; 1.40)	0.3 (-1.2; 1.7)	0.2 (-1.3; 1.6)
Persistent Deprivation	1.72 (1.29; 2.31)	1.54 (1.14; 2.09)	2.8 (1.1; 4.5)	2.3 (0.5; 4.1)	0.95 (0.69; 1.31)	0.87 (0.62; 1.22)	-0.3 (-1.9; 1.3)	-0.7 (-2.4; 1.0)
Loss/Threat of Loss	1.56 (1.05; 2.32)	1.51 (1.01; 2.25)	2.0 (-0.1; 4.0)	1.8 (-0.3; 3.8)	1.35 (0.92; 1.99)	1.27 (0.86; 1.88)	1.5 (-0.7; 3.8)	1.2 (-1.0; 3.4)
High Adversity	2.27 (1.38; 3.72)	2.12 (1.29; 3.50)	4.6 (0.7; 8.5)	4.3 (0.4; 8.2)	1.16 (0.61; 2.19)	1.11 (0.58; 2.14)	0.7 (-2.8; 4.2)	0.5 (-3.0; 4.1)
	**5-year case fatality** Any cause of death within five years of primary cancer diagnosis							
	285 deaths among 3446 primary cancer cases				282 deaths among 4100 primary cancer cases			
	HR (95% CI)	aHR (95% CI)	HD (95% CI)	aHD (95% CI	HR (95% CI)	aHR (95% CI)	HD (95% CI)	aHD (95% CI
Low Adversity	1 (ref)	1 (ref)	0 (ref)	0 (ref)	1 (ref)	1 (ref)	0 (ref)	0 (ref)
Early Life Deprivation	1.04 (0.75; 1.44)	1.00 (0.72; 1.38)	80 (-592; 753)	-31 (-713; 651)	1.16 (0.86; 1.57)	1.15 (0.85; 1.56)	312 (-335; 959)	288 (-359; 935)
Persistent Deprivation	1.77 (1.31; 2.38)	1.53 (1.13; 2.09)	1530 (621; 2440)	1170 (243; 2100)	1.21 (0.87; 1.67)	1.07 (0.76; 1.53)	398 (-327; 1120)	158 (-599; 915)
Loss/Threat of Loss	1.53 (1.03; 2.26)	1.50 (1.00; 2.24)	1070 (-94.2; 2230)	1000 (-176; 2180)	1.32 (0.89; 1.97)	1.26 (0.83; 1.89)	617 (-363; 1600)	504 (-490; 1500)
High Adversity	1.74 (1.02; 2.98)	1.50 (0.86; 2.59)	1480 (-327; 3290)	1060 (-763; 2880)	1.16 (0.61; 2.20)	1.15 (0.60; 2.22)	310 (-1090; 1710)	367 (-1050; 1780)

a = Adjusted for parental age at birth, parental country of origin, parental history of cancer, being born preterm, being born small for gestational age, and birth year.

Supplementary Table 3. Sensitivity analysis adjusting further for parental education. Hazard ratios (HR), hazard difference (HD) per 100,000 individuals per year and 95% confidence intervals (CI) for cancer incidence, cancer mortality and 5-year case-fatality among 1,194,323 individuals with full covariate information including information about parental education according to trajectory groups of childhood adversity.

	**Men** (n=612,740)				**Women** (n=581,583)			
	**Cancer Incidence** First time hospitalization with a cancer							
	No. of incident cases: 3447				No. of incident cases: 4092			
	HR (95% CI)	aHR (95% CI)	HD (95% CI)	aHD (95% CI)	HR (95% CI)	aHR (95% CI)	HD (95% CI)	aHD (95% CI
Low Adversity	1 (ref)	1 (ref)	0 (ref)	0 (ref)	1 (ref)	1 (ref)	0 (ref)	0 (ref)
Early Life Deprivation	1.03 (0.94; 1.12)	1.04 (0.96; 1.14)	1.5 (-3.0; 6.1)	2.3 (-2.4; 6.9)	0.97 (0.89; 1.05)	0.96 (0.88; 1.04)	-2.2 (-7.5; 3.0)	-2.7 (-8.1; 2.7)
Persistent Deprivation	0.96 (0.87; 1.06)	1.00 (0.91; 1.11)	-2.2 (-7.2; 2.9)	0.0 (-5.4; 5.4)	0.88 (0.80; 0.96)	0.89 (0.81; 0.98)	-9.3 (-15.2; -3.4)	-7.8 (-14.0; -1.6)
Loss/Threat of Loss	1.12 (0.99; 1.27)	1.11 (0.98; 1.25)	6.0 (-0.7; 12.7)	5.2 (-1.5; 12.0)	1.05 (0.94; 1.18)	1.02 (0.91; 1.14)	3.4 (-4.2; 11.0)	0.7 (-7.0; 8.4)
High Adversity	1.13 (0.94; 1.35)	1.17 (0.97; 1.41)	6.6 (-4.0; 17.1)	8.6 (-2.3; 19.4)	1.10 (0.93; 1.31)	1.10 (0.92; 1.31)	7.0 (-6.0; 20.0)	6.8 (-6.6; 20.2)
	**Cancer Mortality** Death due to cancer							
	No. of cancer deaths: 299				No. of cancer deaths: 299			
	HR (95% CI)	aHR (95% CI)	HD (95% CI)	aHD (95% CI	HR (95% CI)	aHR (95% CI)	HD (95% CI)	aHD (95% CI
Low Adversity	1 (ref)	1 (ref)	0 (ref)	0 (ref)	1 (ref)	1 (ref)	0 (ref)	0 (ref)
Early Life Deprivation	1.17 (0.86; 1.60)	1.15 (0.84; 1.58)	0.6 (-0.7; 1.9)	0.5 (-0.8; 1.9)	1.06 (0.79; 1.42)	1.01 (0.74; 1.38)	0.3 (-1.2; 1.7)	0.0 (-1.4; 1.5)
Persistent Deprivation	1.71 (1.27; 2.29)	1.57 (1.15; 2.14)	2.7 (1.0; 4.4)	2.3 (0.6; 4.1)	0.93 (0.67; 1.29)	0.82 (0.58; 1.16)	-0.4 (-2.0; 1.2)	-1.1 (-2.8; 0.6)
Loss/Threat of Loss	1.56 (1.05; 2.33)	1.54 (1.03; 2.29)	2.0 (-0.1; 4.)	1.9 (-0.2; 4.0)	1.35 (0.92; 1.99)	1.24 (0.84; 1.83)	1.55 (-0.665; 3.76)	1.1 (-1.1; 3.3)
High Adversity	2.27 (1.39; 3.73)	2.22 (1.32; 3.72)	4.7 (0.7; 8.6)	4.6 (0.6; 8.6)	1.16 (0.61; 2.20)	1.02 (0.52; 2.00)	0.75 (-2.74; 4.24)	0.1 (-3.6; 3.7)
	**5-year case fatality** Any cause of death within five years of primary cancer diagnosis							
	284 deaths among 3444 primary cancer cases				281 deaths among 4089 primary cancer cases			
	HR (95% CI)	aHR (95% CI)	HD (95% CI)	aHD (95% CI	HR (95% CI)	aHR (95% CI)	HD (95% CI)	aHD (95% CI
Low Adversity	1 (ref)	1 (ref)	0 (ref)	0 (ref)	1 (ref)	1 (ref)	0 (ref)	0 (ref)
Early Life Deprivation	1.04 (0.75; 1.44)	0.99 (0.71; 1.38)	80 (-592; 753)	-53 (-745; 638)	1.16 (0.86; 1.58)	1.13 (0.82; 1.56)	317 (-330; 964)	251 (-427; 929)
Persistent Deprivation	1.74 (1.29; 2.35)	1.48 (1.08; 2.04)	1480 (576; 2380)	1070 (141; 2000)	1.19 (0.86; 1.66)	1.02 (0.71; 1.48)	367 (-358; 1090)	47 (-728; 824)
Loss/Threat of Loss	1.53 (1.03; 2.27)	1.50 (1.00; 2.25)	1070 (-94.2; 2230)	984 (-196; 2160)	1.33 (0.89; 1.98)	1.23 (0.82; 1.86)	628 (-354; 1610)	464 (-532; 1460)
High Adversity	1.74 (1.02; 2.98)	1.43 (0.81; 2.53)	1490 (-317; 3300)	959 (-885; 2800)	1.19 (0.63; 2.26)	1.11 (0.57; 2.18)	367 (-1070; 1800)	293 (-1190; 1770)

a = Adjusted for parental age at birth, parental country of origin, parental history of cancer, being born preterm, being born small for gestational age, birth year and parental education.

Supplementary Figure 4. Sensitivity analysis: restricted to a subpopulation without parental history of cancer. Adjusted hazard ratios (aHR) and 95% CI for cancer incidence, cancer mortality and 5-year case-fatality.

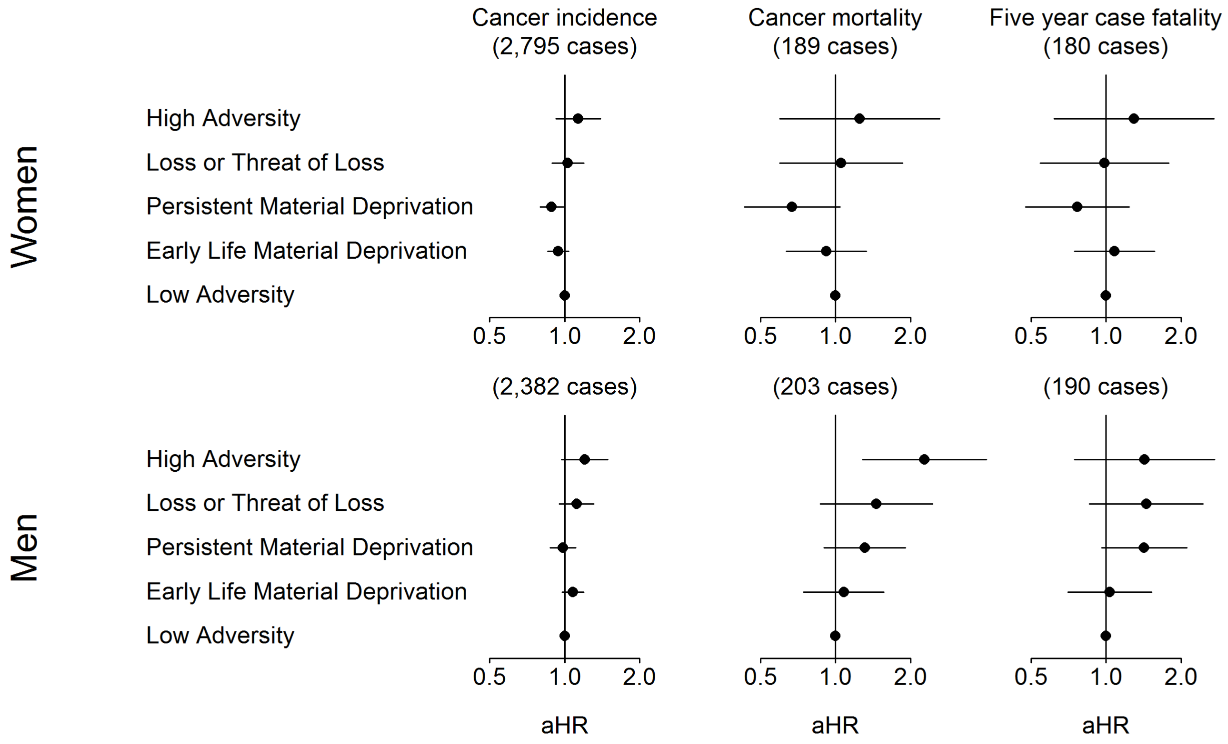

Supplementary Table 5. Unadjusted hazard ratio (HR) and hazard difference (HD) per 100,000 individuals per year for cancer incidence according to childhood adversity trajectories among 582,595 young women and 613,894 young men.

Women

	Malignant melanoma (n=1096)			Breast cancer (n=465)			Brain and CNS (n=689)			Cervical cancer (n=470)		
	No cases	HR (95% CI)	HD (95% CI)	No cases	HR (95% CI)	HD (95% CI)	No cases	HR (95% CI)	HD (95% CI)	No cases	HR (95% CI)	HD (95% CI)
Low Adversity	620	1 (ref)	0 (ref)	209	1 (ref)	0 (ref)	355	1 (ref)	0 (ref)	204	1 (ref)	0 (ref)
Early Life Deprivation	212	0.81 (0.69; 0.94)	-3.8 (-6.5; -1.1)	90	1.04 (0.81; 1.34)	0.3 (-1.4; 2.0)	158	1.07 (0.89; 1.30)	0.8 (-1.4; 3.1)	105	1.20 (0.95; 1.51)	1.3 (-0.5; 3.1)
Persistent Deprivation	148	0.68 (0.57; 0.81)	-6.9 (-9.8; -3.9)	103	1.20 (0.95; 1.52)	1.6 (-0.7; 3.8)	84	0.71 (0.56; 0.90)	-3.5 (-5.7; -1.3)	96	1.23 (0.96; 1.56)	1.6 (-0.5; 3.8)
Loss/Threat of Loss	86	0.87 (0.69; 1.09)	-2.5 (-6.3; 1.3)	42	1.31 (0.94; 1.83)	2.0 (-0.6; 4.5)	67	1.17 (0.90; 1.52)	1.9 (-1.4; 5.2)	42	1.32 (0.95; 1.84)	2.0 (-0.6; 4.6)
High Adversity	30	0.81 (0.56; 1.17)	-3.8 (-9.9; 2.4)	21	1.66 (1.06; 2.60)	4.6 (-0.5; 9.6)	25	1.21 (0.80; 1.81)	2.4 (-3.2; 7.9)	23	1.84 (1.20; 2.84)	5.7 (0.5; 11.0)

Men

	Testicular cancer (n=1079)			Malignant melanoma (n=481)			Brain and CNS (n=569)			Hodgkin's lymphoma (n=255)		
	No cases	HR (95% CI)	HD (95% CI)	No cases	HR (95% CI)	HD (95% CI)	No cases	HR (95% CI)	HD (95% CI)	No cases	HR (95% CI)	HD (95% CI)
Low Adversity	542	1 (ref)	0 (ref)	270	1 (ref)	0 (ref)	274	1 (ref)	0 (ref)	128	1 (ref)	0 (ref)
Early Life Deprivation	240	1.05 (0.90; 1.23)	0.9 (-1.7; 3.4)	91	0.80 (0.63; 1.02)	-1.6 (-3.2; 0.1)	127	1.13 (0.92; 1.40)	1.1 (-0.8; 2.9)	55	1.05 (0.77; 1.44)	0.2 (-1.0; 1.4)
Persistent Deprivation	177	0.96 (0.81; 1.14)	-0.7 (-3.5; 2.2)	68	0.71 (0.54; 0.93)	-2.5 (-4.4; -0.7)	95	1.07 (0.85; 1.35)	0.6 (-1.5; 2.7)	35	0.89 (0.61; 1.29)	-0.4 (-1.7; 0.9)
Loss/Threat of Loss	80	0.94 (0.74; 1.19)	-1.0 (-4.4; 2.5)	38	0.90 (0.64; 1.26)	-0.8 (-3.2; 1.6)	55	1.26 (0.94; 1.70)	2.1 (-0.7; 4.9)	29	1.41 (0.94; 2.11)	1.5 (-0.5; 3.6)
High Adversity	40	1.12 (0.81; 1.55)	2.0 (-3.9; 7.9)	14	0.79 (0.46; 1.34)	-1.7 (-5.3; 1.8)	18	1.02 (0.64; 1.65)	0.2 (-3.8; 4.2)	8	0.98 (0.48; 2.00)	-0.1 (-2.7; 2.6)

Supplementary Table 6. Sensitivity analyses: restricted to a population without parental history of cancer. Adjusted hazard ratio (aHR) and adjusted hazard difference (aHD) per 100,000 individuals per year for cancer incidence according to childhood adversity trajectories among 459,546 young women and 484,900 young men.

Women

	**Malignant melanoma** (n=725)			**Breast cancer** (n=279)			**Brain and CNS** (n=489)			**Cervical cancer** (n=317)		
	No cases	aHR (95% CI)	aHD (95% CI)	No cases	aHR (95% CI)	aHD (95% CI)	No cases	aHR (95% CI)	aHD (95% CI)	No cases	aHR (95% CI)	aHD (95% CI)
Low Adversity	404	1 (ref)	0 (ref)	135	1 (ref)	0 (ref)	252	1 (ref)	0 (ref)	141	1 (ref)	0 (ref)
Early Life Deprivation	146	0.81 (0.67; 0.98)	-3.4 (-6.3; -0.4)	63	1.04 (0.77; 1.40)	0.3 (-1.5; 2.1)	118	1.05 (0.84; 1.32)	0.6 (-1.9; 3.1)	69	1.03 (0.77; 1.37)	0.2 (-1.8; 2.1)
Persistent Deprivation	107	0.77 (0.62; 0.96)	-4.3 (-7.8; -0.9)	52	0.96 (0.69; 1.32)	-0.4 (-2.6; 1.8)	65	0.81 (0.62; 1.07)	-2.0 (-4.6; 0.6)	68	1.21 (0.90; 1.62)	1.5 (-1.0; 4.0)
Loss/Threat of Loss	48	0.93 (0.69; 1.25)	-1.3 (-5.8; 3.3)	13	0.78 (0.44; 1.39)	-1.1 (-3.5; 1.3)	39	1.12 (0.80; 1.57)	1.3 (-2.7; 5.3)	25	1.37 (0.89; 2.09)	2.1 (-1.1; 5.3)
High Adversity	20	0.83 (0.53; 1.30)	-3.0 (-9.5; 3.6)	16	1.99 (1.18; 3.36)	5.7 (0.0; 11.5)	15	0.97 (0.57; 1.63)	-0.4 (-5.9; 5.2)	14	1.51 (0.87; 2.64)	3.4 (-2.0; 8.7)

Men

	**Testicular cancer** (n=749)			**Malignant melanoma** (n=303)			**Brain and CNS** (n=406)			**Hodgkin's lymphoma** (n=188)		
	No cases	aHR (95% CI)	aHD (95% CI)	No cases	aHR (95% CI)	aHD (95% CI)	No cases	aHR (95% CI)	aHD (95% CI)	No cases	aHR (95% CI)	aHD (95% CI)
Low Adversity	372	1 (ref)	0 (ref)	164	1 (ref)	0 (ref)	201	1 (ref)	0 (ref)	95	1 (ref)	0 (ref)
Early Life Deprivation	177	1.09 (0.91; 1.31)	1.4 (-1.5; 4.3)	61	0.83 (0.62; 1.12)	-1.1 (-2.8; 0.6)	96	1.13 (0.88; 1.44)	1.0 (-1.1; 3.1)	47	1.19 (0.83; 1.71)	0.7 (-0.8; 2.2)
Persistent Deprivation	131	1.07 (0.87; 1.31)	1.1 (-2.3; 4.4)	45	0.79 (0.57; 1.11)	-1.5 (-3.6; 0.5)	60	0.98 (0.73; 1.32)	-0.2 (-2.5; 2.2)	25	0.84 (0.52; 1.34)	-0.6 (-2.1; 0.9)
Loss/Threat of Loss	40	0.84 (0.60; 1.17)	-2.2 (-6.2; 1.7)	24	1.17 (0.76; 1.79)	1.0 (-2.0; 4.0)	35	1.35 (0.94; 1.94)	2.7 (-0.9; 6.2)	15	1.15 (0.66; 1.99)	0.6 (-1.8; 2.9)
High Adversity	29	1.19 (0.81; 1.75)	2.9 (-3.8; 9.5)	9	0.84 (0.42; 1.67)	-1.0 (-4.8; 2.8)	14	1.10 (0.64; 1.89)	0.8 (-3.8; 5.4)	6	1.02 (0.44; 2.35)	0.1 (-2.9; 3.1)

a: Adjusted for parental age at birth, parental country of origin, family history of cancer, being born preterm, being born small for gestational age, and birth year.

Supplementary Methods. Group-based multi-trajectory model

We used a group-based multi-trajectory model following the approach in Nagin DS, Jones BL, Passos VL, & Tremblay RE (2018): Group-based multi-trajectory modeling. Statistical methods in medical research, 27(7), 2015-2023. Our intention was to determine the most common trajectories of childhood adversities based on the three predefined dimensions: material deprivation, loss or threat of loss, and family dynamics. For each child, we summed the number of annual adverse childhood events within the three dimensions as illustrated in the hypothetical example below depicting one imaginary child:

Hypothetical example of one study participant

**Dimension**	**Event type**	**Age 0-1**	**Age 1-2**	**…**	**Age 15-16**
Material deprivation	Family poverty	1	0		1
	Long-term unemployment	0	2		1
**Sum vector for dimension 1**		**1**	**2**		**2**
Loss or threat of loss	Death of a parent	0	0		0
	Death of a sibling	0	1		0
	Parental somatic illness	1	2		0
	Sibling somatic illness	1	0		0
**Sum vector for dimension 2**		**2**	**3**		**0**
Family dynamics	Foster care	0	0		0
	Parental psychiatric illness	0	0		0
	Sibling psychiatric illness	0	0		0
	Parental alcohol abuse	0	0		0
	Parental drug abuse	0	0		0
	Maternal separation				
**Sum vector for dimension 3**		**0**	**0**		**0**

As outlined above, each adversity only counts in the years in which it occurs, but the same type of adversity can occur multiple times during childhood. As an example, see the cell in column "Age 1-2" row "Parental somatic illness". Here, having a mother with cancer will count towards the 'loss or threat of loss' dimension only in the years in which the mother has a cancer diagnosis in the hospital register. Additionally, having two parents with cancer the same year will count twice in the giving year. Thus, this imaginary participant has two parents with hospital contacts for severe illnesses during his/her second year of live.

We fit the group-based multi-trajectory models using zero-inflated Poisson regressions that models the trajectories with a cubic function of age. The model specifications can be seen below, as for each individual i, let $D1_i$ denote the vector of summed count values of the material deprivation dimension, $D2_i$ denote the vector of summed count values of the loss or threat of loss dimension, $D3_i$ denote the vector of summed count values of the family dynamics dimension all by age $j \in (0, \dots, 15)$, where j=0 indicates an age bin from 0-1 year and j=15 indicates an age bin from 15 to 16 years. $d1_{ij}$, $d2_{ij}$ and $d3_{ij}$ denotes the observed counts of the respective dimensions for individual, i, and age bin, j. $t \in (1, \dots, 5)$ denotes the latent trajectory group.

$$\Pr(D1_{ij} = 0 | T_i = t) = \pi_{jt} + (1 - \pi_{jt})e^{-\lambda_{jt}} \qquad \text{if } d1_{ij} = 0$$

$$\Pr\left(D1_{ij} = d1_{ij} \mid T_i = t\right) = \left(1 - \pi_{jt}\right) \frac{\lambda_{jt}^{d1_{ij}} e^{-\lambda_{jt}}}{d1_{ij}!} \qquad \text{if } d1_{ij} \geq 1$$

Where

$$\log\left(\lambda_{jt}\right) = \beta_{0t} + \beta_{1t} \cdot j + \beta_{2t} \cdot j^2 + \beta_{3t} \cdot j^3$$

and

$$\log\left(\pi_{jt}/\left(1 - \pi_{jt}\right)\right) = \alpha_{0t} + \alpha_{1t} \cdot j$$

We used the package TRAJ for Stata to fit the trajectories. Due to computational issues with large data, we employed a 2-stage approach. First, we fitted a model based on a random sample of 50,000 individuals and, second, we extrapolated the estimated probabilities of being in each trajectory group onto the full cohort. To assess the consistency of estimating the model on a sub-sample, we re-ran the procedure on 5 random samples of 50,000 individuals selected from the total study population, which returned five almost identical estimated models.

We report our approach using the GRoLTS-Checklist (van de Schoot R, Sijbrandij M, Winter SD, Depaoli S, Vermunt JK. The GRoLTS-Checklist: Guidelines for Reporting on Latent Trajectory Studies. Struct Equ Model 2017; 24: 451–67.)

**GRoLTS-Checklist**	
1. Is the metric of time used in the statistical model reported?	Yes, childhood adversities are coded with 1 year time ranges from 0 years to and with 15 years of age.
2. Is information presented about the mean and variance of time within a wave?	Yes, we show rates by age (means of events by time) by each dimension.
3a. Is the missing data mechanism reported?	Missing data is reported and the distribution of missing data is presented by trajectory group. Missing data was not an exclusion criteria and main results are not adjusted for such co-variates.
3b. Is a description provided of what variables are related to attrition/missing data?	
3c. Is a description provided of how missing data in the analyses were dealt with?	
4. Is information about the distribution of the observed variables included?	Yes, but not in this paper. They can be found in Bengtsson J, Dich N, Rieckmann A, Rod NH. Cohort profile: the DANish LIFE course (DANLIFE) cohort. BMJ Open 2019; 9: e027217.
5. Is the software mentioned?	Yes, we used the package TRAJ for Stata to fit between 1 and 8 trajectory groups using zero inflated Poisson regressions and a quadratic trajectory giving us a probability of being in each trajectory. By visually assessing the trajectories, we judged that 5 trajectories to be optimal.
6a. Are alternative specifications of within-class heterogeneity considered (e.g., LGCA vs. LGMM) and clearly documented? If not, was sufficient justification provided as to eliminate certain specifications from consideration?	
6b. Are alternative specifications of the between-class differences in variance–covariance matrix structure considered and clearly documented? If not, was sufficient justification provided as to eliminate certain specifications from consideration?	
7. Are alternative shape/functional forms of the trajectories described?	
8. If covariates have been used, can analyses still be replicated?	
9. Is information reported about the number of random start values and final iterations included?	No, we do not report the random initiation values or the number of iterations, but we have ensured that the groups are consistently found when the procedure is reproduced on sub samples.
10. Are the model comparison (and selection) tools described from a statistical perspective?	Further information about the approach can be found in Nagin DS, Jones BL, Passos VL, Tremblay RE. Group-based

	multi-trajectory modeling. Stat Methods Med Res 2018; 27: 2015–23.
11. Are the total number of fitted models reported, including a one-class solution?	Yes, we fitted 1 to 8 trajectories and judged that 5 trajectories were optimal.
12. Are the number of cases per class reported for each model (absolute sample size, or proportion)?	Yes.
13. If classification of cases in a trajectory is the goal, is entropy reported?	No.
14a. Is a plot included with the estimated mean trajectories of the final solution?	Yes, this is shown in Figure 2 in the manuscript.
14b. Are plots included with the estimated mean trajectories for each model?	This can be found in the Supplementary material to Rod NH, Bengtsson J, Budtz-Jørgensen E, et al. Trajectories of childhood adversity and mortality in early adulthood: a population-based cohort study. Lancet 2020.
14c. Is a plot included of the combination of estimated means of the final model and the observed individual trajectories split out for each latent class?	No.
15. Are characteristics of the final class solution numerically described (i.e., means, SD/SE, n, CI, etc.)?	We show class solution by means in Figure 2 in the manuscript.
16. Are the syntax files available (either in the appendix, supplementary materials, or from the authors)?	We describe how the multivariate version of the TRAJ package for Stata was operationalized in the Technical appendix to Rod NH, Bengtsson J, Budtz-Jørgensen E, et al. Trajectories of childhood adversity and mortality in early adulthood: a population-based cohort study. Lancet 2020.
