# Supplementary material for: Cancer burden among adolescents and young adults in relation to childhood adversity: a nationwide life-course cohort study of 1.2 million individuals
Source: Lancet Reg Health Eur. 2023 Feb 10;27:100588. doi: 10.1016/j.lanepe.2023.100588 (PMC9945708; doi:10.1016/j.lanepe.2023.100588)
Supplement: ABSTRACT [file mmc2.docx]

The following translation in Danish was submitted by the authors and we reproduce them as supplied. They have not been peer reviewed. Our editorial processes have only been applied to the original abstract in English, which should serve as reference for this manuscript.

ABSTRACT (in Danish)

**Baggrund:** Store stressbelastninger i barndommen, såsom fattigdom, at miste en forælder, eller hvis man vokser op i en dysfunktionel familie, kan muligvis være forbundet med en forstyrrelse af den normale biologisk funktion samt udsættelse for risikofaktorer, som kan påvirke risikoen for at udvikle cancer eller overlevelse efter en cancerdiagnose. For at udforske denne hypotese undersøger vi risikoen for cancer blandt unge mænd og kvinder, der har været udsat for store stressbelastninger i barndommen.

**Metoder:** Hypotesen testes ved brug af danske landsdækkende registerdata om stressbelastninger i barndommen og cancerdiagnoser. Vi inkluderede alle børn, der var født i Danmark og levede i Danmark indtil deres 16-års fødselsdag. Disse børn blev fulgt ind i voksenalderen (16 til 38 år). Ud fra en statistisk clusteranalyse kunne vi kategorisere individerne i fem forskellige grupper: lav stressbelastning, tidlige økonomiske afsavn, vedvarende økonomiske afsavn, tab/trussel om tab og høj stressbelastning. Vi vurderede sammenhængen mellem disse grupper og cancerincidens, dødelighed og femårs-overlevelse efter cancerdiagnose. Desuden undersøgte vi risikoen for de fire mest almindelige cancerformer i denne aldersgruppe i kønsstratificerede overlevelsesanalyser.

**Resultater:** I alt 1.281.334 individer født mellem 1. januar 1980 og 31. december 2001 blev fulgt op indtil 31. december 2018, hvilket inkluderede 8229 incidente cancertilfælde samt 662 cancerdødsfald. Sammenlignet med lav stressbelastninger havde kvinder, der oplevede vedvarende økonomiske afsavn, en lidt lavere risiko for at udvikle cancer (hazard ratio (HR) 0,90; 95% CI 0,82; 0,99), især som følge af malignt melanom og cancer i hjernen og centralnervesystemet, mens kvinder, som havde oplevet høj stressbelastning i barndommen, havde en højere risiko for at udvikle brystcancer (HR 1,71; 95 % CI 1,09; 2,70) og livmoderhalskræft (HR 1,82; 95 % CI 1,18; 2,83). Der var ikke var nogen klar sammenhæng mellem stressbelastning i barndommen og cancerforekomst hos mænd. Til gengæld havde de mænd, der havde oplevet vedvarende økonomiske afsavn (HR 1,72; 95 % CI 1,29; 2,31) eller høj stressbelastning i barndommen (HR 2,27; 95 % CI 1,38; 3,72) en uforholdsmæssig stor byrde af cancerdødelighed i den unge voksenalder sammenlignet med mænd med lav stressbelastning.

**Fortolkning:** Store stressbelastninger barndommen er forbundet med en lavere risiko for udvikling af nogle undertyper af cancer og en højere risiko for andre, især hos kvinder. Vedvarende økonomiske afsavn og stressbelastninger er desuden forbundet med en højere risiko for død efter en cancerdiagnose blandt mænd. Disse resultater skyldes sandsynligvis en kombination af biologisk sårbarhed, sundhedsadfærd og behandlingsrelaterede faktorer.

**Finansiering:** Der er ingen specifik finansiering af studiet.
